# Case Report: Giant anterior-wall uterine leiomyoma with cystic hydropic degeneration mimicking malignancy

**DOI:** 10.3389/fmed.2026.1964095

**Published:** 2026-10-27

**Authors:** Savitri M. Nerune, Sayandeep K. Das, Shailesh Kannur, Ushe Kalyani

**Affiliations:** 1 Department of Pathology, Shri B. M. Patil Medical College Hospital and Research Centre, BLDE (Deemed to be University), Vijayapura, Karnataka, India; 2 Department of Surgery, Shri B. M. Patil Medical College Hospital and Research Centre, BLDE (Deemed to be University), Vijayapura, Karnataka, India

**Keywords:** case report, cystic hydropic degeneration, frozen section, leiomyosarcoma mimic, uterine leiomyoma

## Abstract

**Background:**

Giant uterine leiomyomas with extensive cystic and hydropic degeneration are uncommon and can resemble uterine sarcoma or an adnexal malignancy clinically, radiologically and intraoperatively.

**Case presentation:**

A woman in her 30s presented with six months of abdominal pain and progressive abdominal enlargement, followed by 15 days of bilateral pitting lower-limb oedema. Examination revealed a firm abdominopelvic mass corresponding to a 28–32-week gravid uterus. Contrast-enhanced computed tomography demonstrated an 18.8 × 12.0 × 19.1 cm complex solid-cystic lesion arising from the anterior uterine wall, with irregular enhancing septa, compression of the common iliac vessels and lower inferior vena cava, and distal ureteric compression causing mild hydroureteronephrosis. Leiomyoma with cystic degeneration was favoured, but leiomyosarcoma remained a radiological differential diagnosis. Exploratory laparotomy with tumour excision, hysterectomy, bilateral salpingo-oophorectomy and pelvic lymph-node dissection was performed. The encapsulated mass measured 22 × 20 × 10 cm at final gross examination and released approximately 3 L of blood-stained serous fluid. Frozen section showed a bland spindle-cell smooth-muscle tumour with extensive hyalinisation and cystic change; because of the abnormal gross appearance and limited sampling, a guarded impression favouring “atypical leiomyoma” was issued pending permanent sections. Extensive permanent sampling demonstrated interlacing fascicles of bland smooth-muscle cells with cystic hydropic degeneration and hyalinisation, without significant cytological atypia, mitotic figures or coagulative tumour-cell necrosis. The final diagnosis was giant leiomyoma with cystic hydropic degeneration and hyalinisation. At the 3-month follow-up, the patient remained well without clinical evidence of recurrence.

**Conclusion:**

Marked degeneration, blood-stained serous fluid and major pressure effects can make a benign leiomyoma appear malignant. Frozen-section uncertainty alone is not a justification for radical surgery. Integrated clinical, radiological and histopathological assessment should guide management, with particular attention to fertility and ovarian preservation in young women.

## 1 Introduction

Uterine leiomyomas are the most common benign neoplasms of the female genital tract. Their reported prevalence varies widely according to population and method of detection, and clinically significant tumours may cause abnormal bleeding, pelvic pain, abdominal distension, infertility or pressure symptoms (1). Secondary degeneration can substantially alter their usual firm, whorled appearance. Hyaline change is frequent, whereas extensive hydropic and cystic degeneration is less common and may produce a heterogeneous or predominantly cystic mass with irregular septa and non-enhancing regions on imaging (2).

Giant degenerative leiomyomas may therefore be mistaken for leiomyosarcoma or an adnexal malignancy, particularly when uterine origin is difficult to establish or the lesion produces haemorrhage, necrosis-like areas and major mass effect (3–5). Here, “giant” is used descriptively, as in the case-report literature (3, 4), rather than as a separate histopathological entity; no World Health Organization (WHO)-specific size criterion is being applied. The intraoperative distinction can be equally challenging because frozen sections provide limited sampling of a large, heterogeneous tumour. We report a giant anterior-wall leiomyoma with extensive cystic hydropic degeneration, approximately 3 L of blood-stained serous fluid, iliocaval and ureteric compression, and a guarded frozen-section diagnosis that was resolved by extensive permanent histopathological examination.

## 2 Case presentation

An unmarried woman in her 30s presented with abdominal pain of 6 months’ duration, associated with progressive abdominal fullness and bilateral lower-limb swelling for 15 days, more pronounced on the left side. She had first noticed a lower abdominal mass 6 months earlier. The mass had developed insidiously, was initially small and gradually increased to its current size. The lower-limb swelling was diffuse, pitting in nature, and not associated with pain. There was no history of fever, constipation, vomiting, or trauma. She had no known history of diabetes mellitus, hypertension, tuberculosis, or asthma. Menstrual history revealed menarche at 15 years of age with irregular cycles for the past 10 years. Family history was non-contributory.

On general physical examination, she was moderately built and nourished, afebrile, and haemodynamically stable, with a pulse rate of 80 beats/min, blood pressure of 110/70 mmHg, and oxygen saturation of 98% on room air. There was no pallor, icterus, cyanosis, clubbing, or lymphadenopathy. Bilateral pitting pedal oedema was present.

Abdominal examination revealed fullness of the abdomen with stretched overlying skin and an everted, raised umbilicus. A single large abdominal mass was noted, extending from 10 cm below the xiphisternum to the pubic symphysis, corresponding clinically to the size of a 28–32-week gravid uterus. On palpation, the mass was solitary, globular, and approximately 20 × 16 cm in size, with well-defined borders except inferiorly. It was hard in consistency and horizontally mobile, with mild to moderate tenderness on deep palpation. Percussion revealed a dull note over the mass, and bowel sounds were obscured on auscultation due to the large lesion.

Contrast-enhanced computed tomography (CT) of the abdomen and pelvis demonstrated a large cystic lesion occupying the pelvis and lower abdomen, measuring approximately 18.8 × 12.0 × 19.1 cm. The lesion showed a thick enhancing wall with multiple thick and thin irregular internal septa and appeared to arise from the anterior wall of the uterus, which formed a claw-like configuration around the lesion. The mass produced significant pressure effects on adjacent structures, including superior displacement and crowding of bowel loops, rightward displacement of the urinary bladder, compression of bilateral common iliac vessels and the lower inferior vena cava, and compression of both distal ureters resulting in mild hydroureteronephrosis. Mild free fluid was noted in the pelvis. Radiologically, the lesion was considered to be of uterine origin, most likely a leiomyoma with cystic degeneration; however, because of its large size and atypical imaging features, leiomyosarcoma was reported as a differential diagnosis. Multiple additional intramural uterine fibroids were present, the largest measuring 2.5 × 2.4 cm. No enlarged pelvic lymph nodes were reported on CT.

No preoperative biopsy was performed. The treating team proceeded to operative assessment because of the marked pressure effects and radiological concern for malignancy, without preoperative histological confirmation.

In view of the giant abdominopelvic mass with marked compressive effects on adjacent viscera and vessels, and the radiological concern for an atypical uterine neoplasm, the patient underwent exploratory laparotomy with intraoperative frozen-section assessment. The completed procedure included excision of the tumour with hysterectomy, bilateral salpingo-oophorectomy, and pelvic lymph-node dissection under spinal anaesthesia. The decision to perform bilateral salpingo-oophorectomy was made after the guarded frozen-section impression favouring “atypical leiomyoma”, which the treating team considered together with the radiological concern, large tumour size and blood-stained fluid. Pelvic lymph-node dissection was undertaken because of the same persisting concern for malignancy despite the absence of enlarged nodes on CT. The frozen-section impression did not establish a diagnosis of leiomyosarcoma. The resected specimen was subsequently submitted for pathological evaluation.

### 2.1 Frozen section and histopathological findings

Intraoperative frozen-section examination was performed on the mass arising from the anterior uterine wall. Grossly, the specimen consisted of a large encapsulated cystic and solid tumour measuring 22 × 20 × 10 cm. The external surface was smooth and glistening, with no evidence of capsular breach. On cut section, approximately 3 L of blood-stained serous fluid was drained. The lesion was predominantly solid with focal cystic areas (Figure 1).

**Figure 1:**
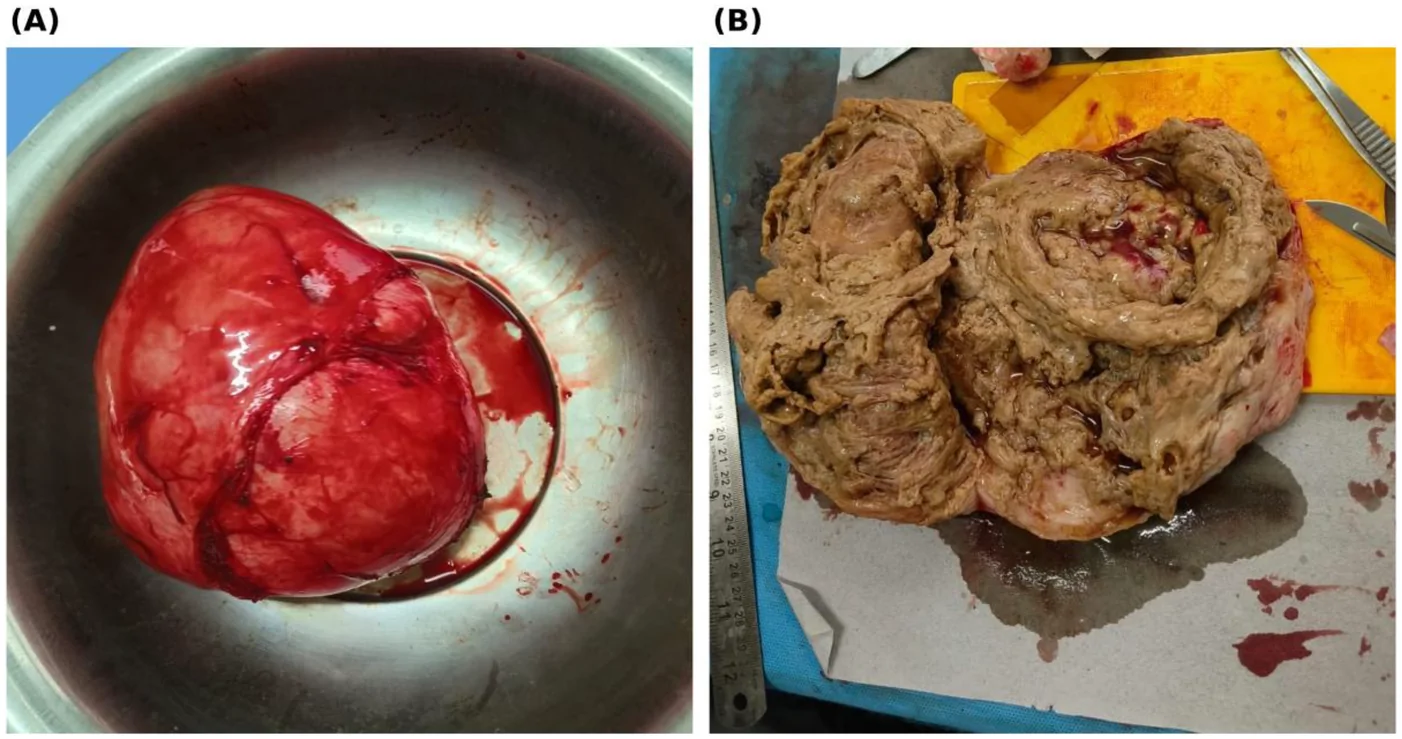
Gross pathological appearance. **(A)** Encapsulated uterine mass with a smooth, glistening external surface and prominent surface vessels. **(B)** Cut surface showing a predominantly solid whorled lesion with extensive pale hyalinised and cystic-degenerative areas.

Microscopically, the frozen section showed a spindle-cell smooth-muscle tumour arranged in interlacing fascicles and bundles. The individual tumour cells were elongated, with blunt-ended nuclei and a moderate amount of eosinophilic cytoplasm. Large areas of hyalinisation were noted, along with cystic degenerative change and scattered haemosiderin-laden macrophages. On the frozen sections examined, significant cytological atypia, abnormal or atypical mitotic figures, brisk mitotic activity, bizarre multinucleated tumour cells and coagulative tumour-cell necrosis were not identified. However, in view of the unusually large size of the tumour, the complex solid-cystic gross appearance, drainage of approximately 3 L of blood-stained serous fluid, the radiological differential diagnosis of leiomyosarcoma, and the limited representativeness of frozen-section sampling, a cautious intraoperative impression of a smooth-muscle tumour, favouring “atypical leiomyoma” with cystic degeneration and extensive hyalinisation, was rendered. Correlation with adequately sampled permanent histopathological sections was advised for definitive classification and to rule out malignancy.

On final histopathological examination, the specimen was received in four containers and comprised the uterus with tumor and pelvic lymph nodes. The anterior uterine wall mass was encapsulated. Following the drainage described above, the cut surface was predominantly solid with a characteristic whorled appearance and focal cystic change. The uterocervix with bilateral adnexa measured 9 × 6.5 × 5 cm. Four subserosal nodules were also identified, the largest measuring 2 × 1 cm. Bilateral ovaries and fallopian tubes were grossly unremarkable. Three pelvic lymph node specimens were also submitted separately.

Microscopically, sections from the anterior uterine wall mass showed a spindle-cell neoplasm composed of elongated cells arranged in interlacing sheets, bundles, and whorls. The cells had elongated nuclei with blunt ends and moderate eosinophilic cytoplasm. Extensive hyalinization and cystic hydropic degeneration were present, with loose oedematous stroma and congested, dilated blood vessels. Extensive sampling of the mass showed no significant nuclear atypia or coagulative tumour-cell necrosis (Figures 2, 3). Mitotic figures were absent in the examined permanent sections. The number of high-power fields examined and the evaluated field area were not available in the records reviewed. Immunohistochemistry was not performed because the permanent-section morphology was considered characteristic of a degenerative leiomyoma.

**Figure 2:**
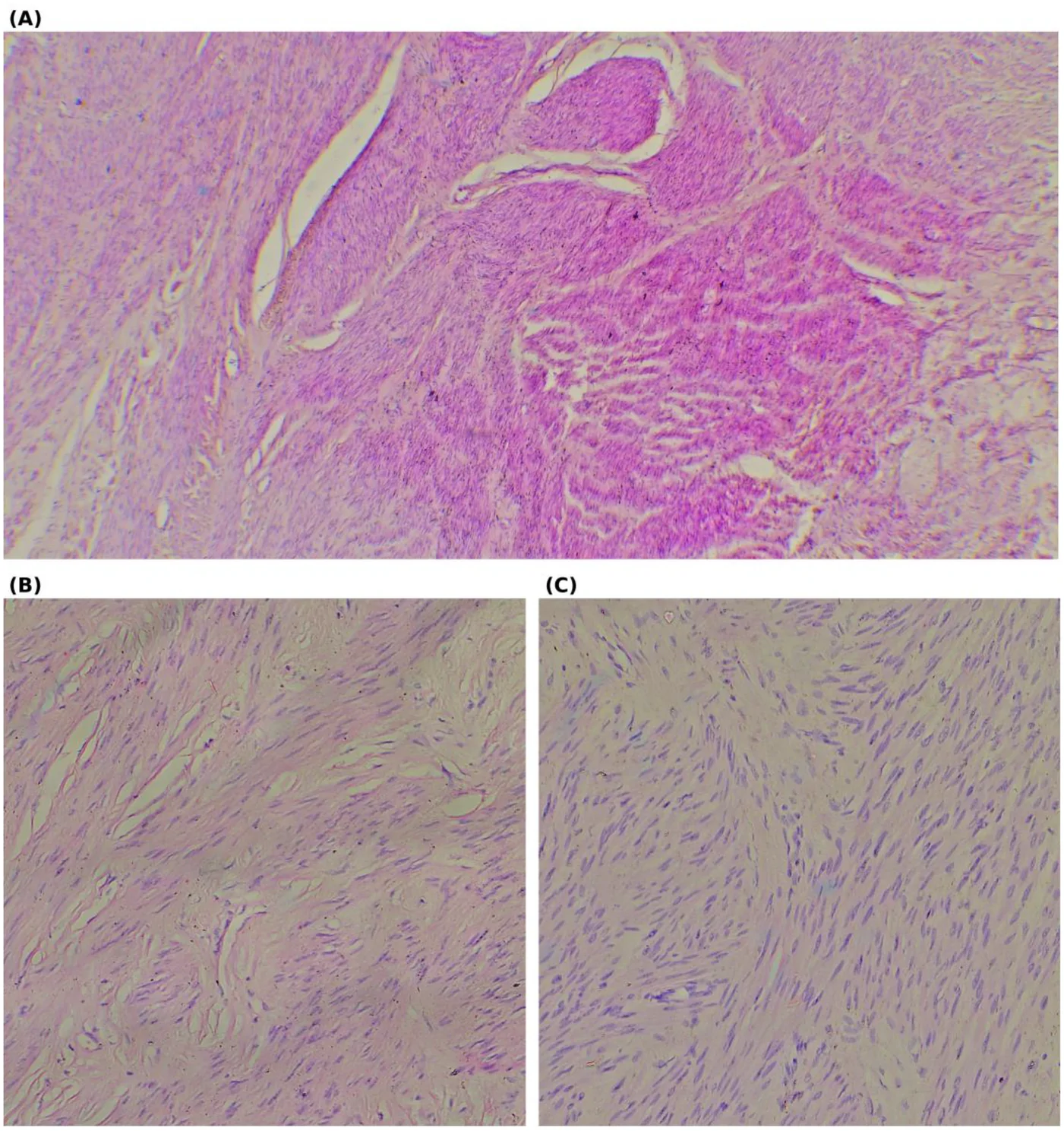
Permanent-section histopathology showing smooth-muscle morphology. **(A)** Low-power view demonstrating intersecting and whorled smooth-muscle fascicles. **(B)** Intermediate-power view showing bland spindle-cell fascicles separated by hyalinised and hydropic stroma. **(C)** Higher-power view showing elongated spindle cells with blunt-ended nuclei and eosinophilic cytoplasm, without significant cytological atypia. Haematoxylin and eosin stain. Objective magnifications were not available in the supplied image metadata.

**Figure 3:**
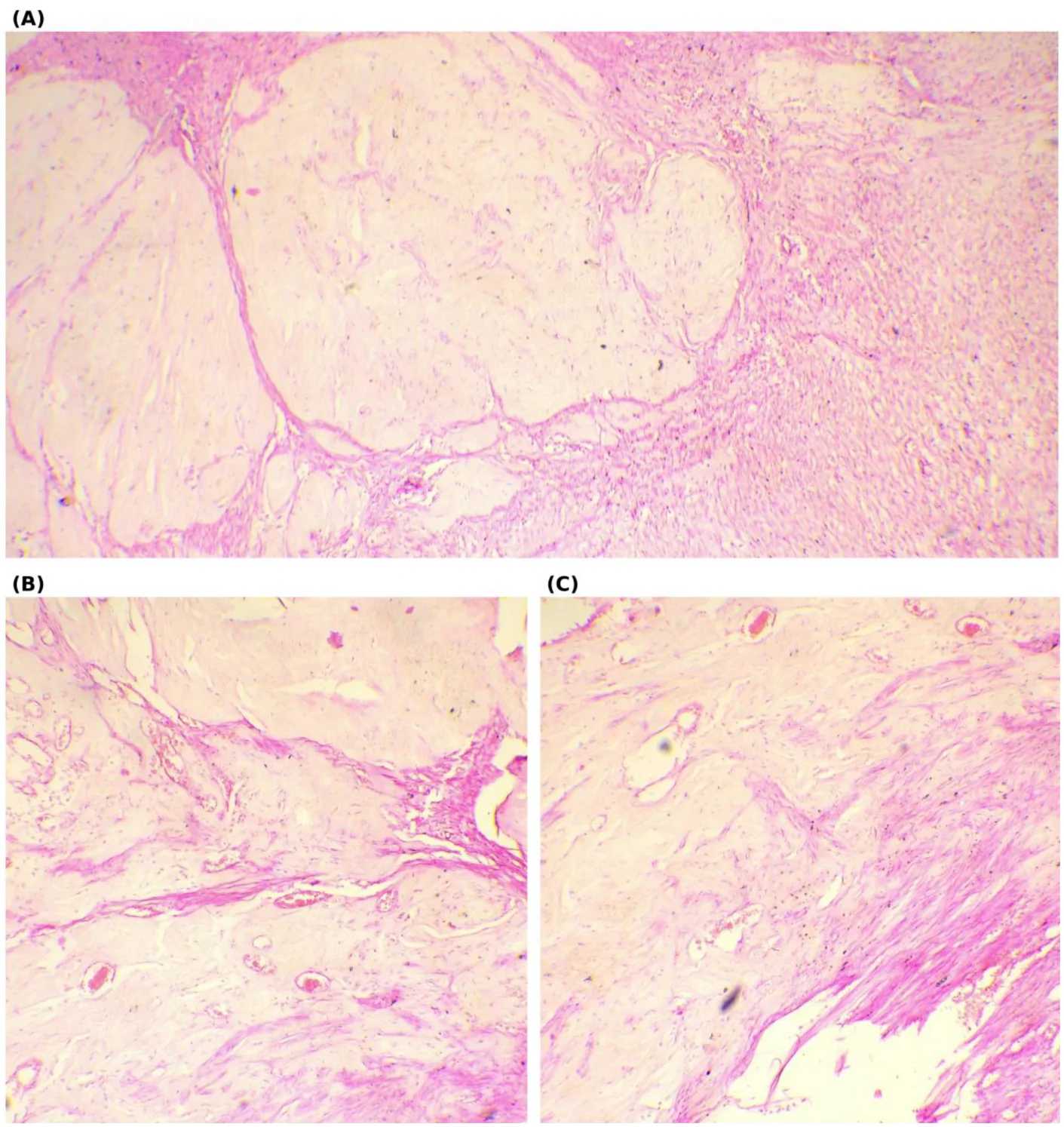
Permanent-section histopathology showing degenerative changes. **(A)** Low-power view demonstrating broad cystic-hydropic and hyalinised zones adjacent to residual smooth-muscle tumour. **(B)** Intermediate-power view showing loose oedematous and hyalinised stroma with congested vessels. **(C)** Higher-power view highlighting the interface between markedly hyalinised stroma and residual spindle-cell tumour. Haematoxylin and eosin stain. Objective magnifications were not available in the supplied image metadata.

Sections from the cervix showed ectocervix lined by stratified squamous epithelium and endocervical glands lined by columnar epithelium, with squamous metaplasia and papillary hyperplasia. The subepithelium showed a chronic inflammatory cell infiltrate. Endometrial sections showed features of secretory-phase endometrium. Sections from the myometrium showed adenomyosis. The subserosal nodules were consistent with leiomyomas. Bilateral adnexa were unremarkable. The right ovary showed a corpus albicans and a corpus hemorrhagicum, while the left ovary showed a corpus albicans and a cystic follicle. Bilateral fallopian tubes showed normal histology. Sections from the pelvic lymph nodes showed no evidence of tumour.

The final histopathological diagnosis was giant leiomyoma of the anterior uterine wall with cystic hydropic degeneration and hyalinisation, without evidence of malignancy. Associated findings included papillary endocervicitis with squamous metaplasia, secretory endometrium, adenomyosis, subserosal leiomyomas, unremarkable bilateral adnexa, and tumour-free pelvic lymph nodes.

### 2.2 Outcome and follow-up

At the documented 3-month follow-up, the patient was clinically well without evidence of recurrence. Follow-up was scheduled every three months (Table 1).

**Table 1:** Timeline of the episode of care.

Time point	Clinical event/finding
Approximately 6 months before admission	Lower abdominal mass first noticed; progressive abdominal fullness and pain began.
15 days before admission	Diffuse bilateral pitting lower-limb oedema developed, more marked on the left.
At admission	Haemodynamically stable; firm abdominopelvic mass equivalent in size to a 28–32-week gravid uterus; bilateral pedal oedema.
Diagnostic imaging	Contrast-enhanced CT showed an 18.8 × 12.0 × 19.1 cm anterior-wall uterine solid-cystic mass, iliocaval compression and mild bilateral hydroureteronephrosis; leiomyosarcoma remained a differential diagnosis. No enlarged pelvic lymph nodes were reported.
Surgery and frozen section	Exploratory laparotomy with tumour excision and frozen-section assessment. The provisional impression favoured “atypical leiomyoma”, with definitive classification deferred. Bilateral salpingo-oophorectomy followed this guarded result and persisting concern for malignancy. The completed operation also included hysterectomy and pelvic lymph-node dissection.
Permanent histopathology	Extensive sampling showed bland interlacing smooth-muscle fascicles with cystic hydropic degeneration and hyalinisation, without significant atypia or coagulative tumour-cell necrosis. Mitotic figures were absent in the examined permanent sections.
Three months after surgery	The patient was clinically well without evidence of recurrence; no follow-up imaging result was documented.

## 3 Discussion

This case illustrates three converging diagnostic pitfalls: extreme tumour size, extensive hydropic-cystic and hyaline degeneration, and clinically important venous and ureteric compression. Degeneration develops when a leiomyoma outgrows or otherwise compromises its blood supply. Hyalinisation may replace broad areas of smooth muscle, while progressive oedema and liquefaction create hydropic and cystic spaces. These changes can obscure the typical firm, whorled phenotype and instead produce irregular septa, fluid-filled cavities, heterogeneous enhancement and apparent necrosis on imaging (2). The anterior myometrial claw in the present case was therefore an important clue to uterine origin, but it did not resolve the benign-versus-malignant question.

Several reports demonstrate how closely this pattern may resemble malignancy. Akkour et al. described a 33-cm leiomyoma with massive cystic hydropic degeneration that clinically and radiologically simulated an aggressive uterine or ovarian tumour (3). Aydin et al. reported a giant pedunculated cystic leiomyoma interpreted preoperatively as ovarian malignancy (4). More recently, a completely cystic submucosal leiomyoma was mistaken for an empty gestational sac until hysteroscopic and pathological assessment established the diagnosis (5). The present tumour adds a different clinicopathological configuration: a documented anterior-wall origin, approximately 3 L of blood-stained serous fluid, extensive hyalinisation and cystic hydropic change, and simultaneous iliocaval and bilateral distal ureteric compression.

The lower-limb oedema was clinically concordant with the imaging evidence of common iliac and inferior vena caval compression. Large fibroids have previously caused mechanical obstruction of the distal inferior vena cava and both ureters, with hydronephrosis and thrombosis (6), and severe lower-extremity oedema due to caval compression has been relieved after treatment of the fibroid burden (7). These observations support a direct mass-effect mechanism in the present patient, although venous thrombosis was not documented in the available clinical records. The combination of pedal oedema and hydroureteronephrosis is therefore not evidence of malignant behaviour; it reflects the anatomical consequences of tumour volume.

Frozen-section consultation for uterine smooth-muscle tumours is uncommon and is most difficult when the usual whorled cut surface is lost, necrosis is uncertain, or there are pronounced stromal alterations. In a 112-case series, abnormal gross appearance was a leading indication for consultation, while stromal alteration, unusual morphology and uncertainty regarding necrosis accounted for most deferrals and diagnostic discrepancies (8). Hyalinisation can be overinterpreted as tumour-cell necrosis on frozen material, and freezing artefact may further impair assessment of nuclear features and mitoses. For a 22-cm heterogeneous tumour, a small number of frozen blocks cannot reliably represent the most worrisome area. A descriptive diagnosis with explicit deferral of definitive classification is therefore safer than upgrading a lesion solely because of size or gross complexity.

The provisional phrase “atypical leiomyoma” also requires context. In this report, it records the guarded intraoperative wording, not a final diagnosis of leiomyoma with bizarre nuclei or a smooth-muscle tumour of uncertain malignant potential. The permanent sections did not show the cytological atypia needed to justify an atypical histological designation. Contemporary classification of conventional spindle-cell uterine smooth-muscle tumours relies principally on the integrated assessment of cytological atypia, mitotic activity and coagulative tumour-cell necrosis (9, 10). Tumour size, haemorrhagic fluid, cystic change and pressure effects may heighten clinical suspicion but are not independently diagnostic of leiomyosarcoma. In this case, extensive permanent sampling consistently demonstrated bland fascicular smooth muscle, no significant atypia, no mitotic figures and no coagulative tumour-cell necrosis, supporting benign leiomyoma.

The permanent sections also helped distinguish degeneration from important mimics. Hydropic change can separate smooth-muscle cells and create a deceptively myxoid or pseudoepithelioid appearance; extensive hyalinisation may resemble necrosis; and congested vessels may draw attention toward an endometrial stromal neoplasm. Correlation with the grossly circumscribed lesion and identification of conventional smooth-muscle fascicles in viable areas are essential. Immunohistochemistry was not undertaken because the permanent sections showed characteristic smooth-muscle morphology with degenerative change. This explains the original diagnostic approach rather than suggesting that ancillary testing has no role. Desmin and h-caldesmon can support smooth-muscle differentiation when lineage is uncertain; p16, p53 and Ki-67 may provide complementary information in equivocal cases, but their expression overlaps between leiomyoma variants and leiomyosarcoma and cannot replace morphological assessment (10, 11).

This case highlights the risk of overtreatment when a benign but atypical uterine mass mimics malignancy. In retrospect, a more complete diagnostic evaluation and explicit consideration of less extensive, fertility- and ovarian-preserving options might have allowed better-tailored management. The operation performed is not presented as a model of recommended care. The favourable short-term outcome does not establish that the surgical extent was necessary.

The operative extent reflected the severe mass effect and concern for a malignant uterine neoplasm, including uncertainty after frozen section. This describes the treating team's reasoning, not evidence that every component of the operation was required. Current uterine-sarcoma guidance does not recommend routine systematic lymphadenectomy when nodes are not suspicious, and ovarian preservation can be considered in selected premenopausal patients with uterus-confined disease (12). A guarded frozen-section result alone should not be treated as a diagnosis of sarcoma or an independent indication for bilateral salpingo-oophorectomy. The available records do not establish whether myomectomy alone was technically feasible, nor do they document reproductive preferences, counselling about uterine and ovarian preservation, or the complete intraoperative decision sequence. These gaps limit retrospective assessment of the surgical extent; no preference for definitive surgery can be inferred from the patient's unmarried status.

Preoperative magnetic resonance imaging (MRI) can improve characterisation of an atypical uterine mass, and specialist image-guided biopsy may be considered in selected circumstances, with recognition of possible false-negative sampling (12). Radiological suspicion is not equivalent to histological confirmation. The decision to proceed without biopsy is therefore reported as part of this case's clinical pathway, not as a recommendation to avoid tissue assessment because its findings might differ from imaging. Where clinically feasible, treatment planning should integrate clinical assessment, specialist imaging, appropriate tissue evaluation and multidisciplinary review before irreversible surgery. In young women, fertility goals, ovarian function, alternatives and the risks of each option require explicit counselling and shared decision-making (12).

The main strength of this report is the clinicoradiological, gross, frozen-section and permanent-section correlation in a lesion that simulated malignancy at multiple stages. It also documents a rare combination of extensive cystic hydropic degeneration, blood-stained serous fluid and major iliocaval and ureteric compression. Limitations include the unavailability of an MRI report, the lack of a documented preoperative laboratory or tumour-marker profile, the unavailable field count and area for mitotic assessment, the short follow-up of 3 months and the absence of a recorded patient perspective. Objective magnification metadata were not available for the submitted photomicrographs; the figures are therefore described as low-, intermediate- and higher-power views rather than assigned unverified objective values.

## 4 Conclusion

A giant leiomyoma with extensive cystic hydropic degeneration and hyalinisation can closely mimic uterine or adnexal malignancy and can produce substantial venous and urinary tract compression. Intraoperative interpretation should be guarded when frozen sections show marked stromal alteration but lack unequivocal atypia, abnormal mitotic activity or coagulative tumour-cell necrosis. Adequate permanent sampling and strict application of established morphological criteria are decisive for avoiding overdiagnosis and inappropriate classification. This report does not advocate radical surgery on the basis of tumour size, radiological suspicion or an indeterminate frozen-section impression alone. Careful clinical, radiological and histopathological assessment should guide an individualised plan that explicitly considers fertility and ovarian preservation in young women.

## 5 Patient perspective

A formal patient perspective was not documented in the available records.

## Data Availability

The original contributions presented in the study are included in the article/Supplementary Material. Further inquiries can be directed to the corresponding author.
